# Characterization of a Lipoyl Domain-independent B-cell Autoepitope on the Human Branched-chain Acyltransferase in Primary Biliary Cirrhosis and Overlap Syndrome with Autoimmune Hepatitis

**DOI:** 10.1080/10446670310001642159

**Published:** 2003

**Authors:** Antal Csepregi, Petra Obermayer-Straub, Susanne Kneip, Anne Kayser, Stephanie Loges, Eleonore Schmidt, Elemér Nemesánszky, Ferenc Szalay, Michael P. Manns, Christian P. Strassburg

**Affiliations:** 1 Department of Gastroenterology Hepatology and Endocrinology Hannover Medical School Carl-Neuberg-Street 1 Hannover 30625 Germany; 2 Department of Gastroenterology and Medicine Polyclinic of the Hospitaller Brothers of St. John of God in Budapest Germany; 3 Department of Gastroenterology Hepatology and Infectious Diseases Otto-von-Guericke University Magdeburg Germany; 4 1st Department of Medicine Semmelweis Medical University Budapest Hungary

## Abstract

*Background and aims*: Antimitochondrial antibodies (AMA) which recognize pyruvate acetyltransferase (PDC-E2) represent a highly diagnostic feature of primary biliary cirrhosis (PBC). The analysis of immunofluorescence (IF)-AMA-positive sera in PBC patients indicates a conformational epitope located within the lipoyl binding domain of bovine branched-chain acyltransferase (BCKADC-E2) alone or in combination with AMA directed against PDC-E2 the significance of which is presently unclear. In the present study, immunoreactivities and disease associations of AMA against BCKADC-E2 were analyzed. B-cell autoepitopes on BCKADC-E2 were mapped by immunoprecipitation assay.

*Methods*: Sera of 96 IF-AMA-positive patients with serological evidence of anti-BCKADC-E2 alone (*n*=26), anti-PDC-E2 alone (*n*=15), and both anti-BCKADC-E2 and anti-PDC-E2 (*n*=55) were analyzed by Western blot and ELISA in addition to an analysis of B cell autoepitopes on BCKADC-E2 by immunoprecipitation using *in vitro* translated, unmodified human proteins. Ninety-four patients without IF-AMA [blood donors (*n*=30), rheumatoid arthritis (*n*=40), autoimmune hepatitis (AIH) (*n*=10) and primary sclerosing cholangitis (PSC) (*n*=14] served as controls.

*Results*: Eighty of 81 (99%) sera positive for BCKADC-E2 recognized the full length, mature protein, while only 2/10 AIH sera and none of the other controls showed reactivity. Of the 68 PBC sera 58 (85%) recognized the N-terminus consisting of aa 1-144 representing the lipoyl domain. Surprisingly, C-terminal sequences (aa 143-421) were recognized by 46 out of 68 sera (68%). Three PBC sera reacted with the C-terminus only. Only 1/7 serum from patients with an “overlap syndrome of PBC and AIH” was reactive with C-terminal sequences.

*Conclusions*: Our analysis of BCKADC-E2-positive PBC sera identified a novel B cell epitope on the C-terminal part of the human protein. Our data indicate that a distinct subset of AMA recognize sequence(s) on BCKADC-E2 which located outside of the lipoyl binding domain. The absence of immunoreactivity against C-terminal sequences may serve as a marker differentiating patients with PBC and overlap syndrome of PBC with AIH.


*Clinical & Developmental Immunology*, June–December 2003 Vol. 10 (2–4), pp. 173–181

Taylor & Francis healthsciences

### Characterization of a Lipoyl Domain-independent B-cell Autoepitope on the Human Branched-chain Acyltransferase in Primary Biliary Cirrhosis and Overlap Syndrome with Autoimmune Hepatitis

ANTAL CSEPREGI[a,b,c], PETRA OBERMAYER-STRAUB[a], SUSANNE KNEIP[a], ANNE KAYSER[a], STEPHANIE LOGES[a], ELEONORE SCHMIDT[a], ELEMÉR NEMESÁNSZKY[b], FERENC SZALAY[d], MICHAEL P. MANNS[a] and CHRISTIAN P. STRASSBURG[a,*]

[a]*Department of Gastroenterology, Hepatology and Endocrinology, Hannover Medical School, Carl-Neuberg-Street 1, 30625 Hannover, Germany;* [b]*Department of Gastroenterology and Medicine, Polyclinic of the Hospitaller Brothers of St. John of God in Budapest Germany;* [c]*Department of Gastroenterology, Hepatology and Infectious Diseases, Otto-von-Guericke University, Magdeburg, Germany;* [d]*1st Department of Medicine, Semmelweis Medical University, Budapest, Hungary*

*Background and aims*: Antimitochondrial antibodies (AMA) which recognize pyruvate acetyltransferase (PDC-E2) represent a highly diagnostic feature of primary biliary cirrhosis (PBC). The analysis of immunofluorescence (IF)-AMA-positive sera in PBC patients indicates a conformational epitope located within the lipoyl binding domain of bovine branched-chain acyltransferase (BCKADC-E2) alone or in combination with AMA directed against PDC-E2 the significance of which is presently unclear. In the present study, immunoreactivities and disease associations of AMA against BCKADC-E2 were analyzed. B-cell autoepitopes on BCKADC-E2 were mapped by immunoprecipitation assay.

*Methods*: Sera of 96 IF-AMA-positive patients with serological evidence of anti-BCKADC-E2 alone ($n = 26$), anti-PDC-E2 alone ($n = 15$), and both anti-BCKADC-E2 and anti-PDC-E2 ($n = 55$) were analyzed by Western blot and ELISA in addition to an analysis of B cell autoepitopes on BCKADC-E2 by immunoprecipitation using *in vitro* translated, unmodified human proteins. Ninety-four patients without IF-AMA [blood donors ($n = 30$), rheumatoid arthritis ($n = 40$), autoimmune hepatitis (AIH) ($n = 10$) and primary sclerosing cholangitis (PSC) ($n = 14$] served as controls.

*Results*: Eighty of 81 (99%) sera positive for BCKADC-E2 recognized the full length, mature protein, while only 2/10 AIH sera and none of the other controls showed reactivity. Of the 68 PBC sera 58 (85%) recognized the N-terminus consisting of aa 1–144 representing the lipoyl domain. Surprisingly, C-terminal sequences (aa 143–421) were recognized by 46 out of 68 sera (68%). Three PBC sera reacted with the C-terminus only. Only 1/7 serum from patients with an "overlap syndrome of PBC and AIH" was reactive with C-terminal sequences.

*Conclusions*: Our analysis of BCKADC-E2-positive PBC sera identified a novel B cell epitope on the C-terminal part of the human protein. Our data indicate that a distinct subset of AMA recognize sequence(s) on BCKADC-E2 which located outside of the lipoyl binding domain. The absence of immunoreactivity against C-terminal sequences may serve as a marker differentiating patients with PBC and overlap syndrome of PBC with AIH.

*Keywords*: Lipoyl domain; Epitope; Primary biliary cirrhosis; Antimitochondrial antibodies; Branched-chain acyltransferase

*Abbreviations*: PBC, primary biliary cirrhosis; AMA, antimitochondrial antibodies; BCKADC-E2, branched-chain acyltransferase; RLA, radioligand assay; PDC-E2, dihydrolipoamide acetyltransferase; pCITE, plasmid vector with cap-independent translation enhancer sequence; cpm, counts per minute; SDS-PAGE, sodium dodecyl sulfate polyacrylamide electrophoresis; AIH, autoimmune hepatitis; PSC, primary sclerosing cholangitis; OLT, orthotopic liver transplantation

### INTRODUCTION

The loss of B-cell tolerance toward mitochondrial antigens is a highly specific serological marker of primary biliary cirrhosis (PBC) present at high titers in patients with PBC (Baum, 1994; Berg and Klein, 1995). PBC is a chronic cholestatic liver disease predominantly affecting middle-aged women. It is characterized by the progressive

*Corresponding author. Tel.: +49-511-532-2853. Fax: +49-511-532-2093. E-mail: strassburg.christian@mh-hannover.de

ISSN 1740-2522 print/ISSN 1740-2530 online © 2003 Taylor & Francis Ltd
DOI: 10.1080/10446670310001642159

destruction of the small intrahepatic bile ducts below 100 μm diameter and by portal inflammation ultimately leading to biliary cirrhosis in a variable course of time. An early diagnosis is required for therapeutic planning and is significantly influenced by the specific detection of antimitochondrial antibodies (AMA) in serum (Walker *et al.*, 1965; Kaplan, 1996; Kim *et al.*, 2000).

The analysis of AMA autoepitopes has led to the identification of a dominant epitope of the major autoantigen for AMA in PBC, the dihydrolipoamide acetyltransferase of the pyruvate dehydrogenase complex (PDC-E2), which is located within a 20 amino acid hydrophilic segment (residues 167–186) around the lipoic acid bound lysine of the inner lipoyl domain (Gershwin *et al.*, 1987; Coppel *et al.*, 1988; van de Water *et al.*, 1988; Surh *et al.*, 1990). This short peptide, although central to the dominant epitope, is a weak antigen. The entire inner lipoyl binding domain (residues 128–221) was demonstrated to be necessary for full immunoreactivity (Burroughs *et al.*, 1992). Use of a cDNA clone isolated from a human fetal liver library showed that a second mitochondrial enzyme of 52 kDa, the branched-chain acyltransferase (BCKADC-E2) was also recognized by AMA from a large number of PBC patients (Surh *et al.*, 1989). Up to 71% of PBC sera were reported to recognize BCKADC-E2, of which 5–20% of sera exclusively reacted with this autoantigen without the presence of anti PDC-E2 antibodies (Fregeau *et al.*, 1989; James *et al.*, 1989; Iwayama *et al.*, 1992; Leung *et al.*, 1995). AMA against BCKADC-E2 were found to inhibit the function of the branched-chain α-keto-acid dehydrogenase enzyme complex (Fregeau *et al.*, 1989).

Available data on B cell autoepitope(s) on the BCKADC-E2 molecule were generated using expression clones of bovine BCKADC-E2 cDNA (Griffin *et al.*, 1990). Leung and colleagues evaluated PBC sera ($n = 9$) and reported that the major epitope resided on the lipoyl binding domain. However, only the full-length clone encoding the mature protein was sufficient to remove all detectable reactivity with BCKADC-E2. Recently, published data suggest that BCKADC-E2 may have further autoepitopes other than the lipoyl domain itself (Migliaccio *et al.*, 1998).

In our analysis, we have used a full-length human cDNA encoding BCKADC-E2 (Lau *et al.*, 1992) and mapped epitope(s) by a radioligand assay (RLA) in order to gain more insight into the nature of the B-cell immune response to BCKADC-E2 and to evaluate the clinical significance of BCKADC-E2 autoantibodies in PBC.

### PATIENTS AND METHODS

### Serum Samples and Patients

Ninety-six sera with a positive immunofluorescence AMA test (IF-AMA) on rat tissue sections (kidney, liver and stomach) or on HEp2 cells at a titer of ≥1:80 were selected for further autoantibody determinations. Twenty-seven sera were obtained from Hungarian patients and 69 sera were selected among sera evaluated in the Autoantibody Laboratory of the Department of Gastroenterology and Hepatology (Medical School of Hannover, Germany). IF-AMA-positive sera were further evaluated by Western blot (WB) and ELISA. Sera giving the classical IF-AMA pattern that were positive for BCKADC-E2 reactivity detected both by WB and ELISA were considered as BCKADC-E2 positive and were selected for epitope mapping. Epitope mapping experiments were performed under blinded conditions, because at the time of autoantibody determinations clinical, biochemical and histological data were not available for investigators in order to exclude bias in the sera analysis.

Controls included 94 sera without IF-AMA obtained from healthy blood donors ($n = 30$), patients with rheumatoid arthritis but without any evidence of liver disease ($n = 40$), autoimmune hepatitis (AIH) ($n = 10$) and primary sclerosing cholangitis (PSC) ($n = 14$). Rheumatoid arthritis, AIH and PSC were diagnosed according to international criteria (Wiesner and La Russo, 1980; Arnett *et al.*, 1988; Johnson and McFarlane, 1992). All sera were stored at −20°C until further analysis. Informed consent was obtained from all patients, and the study protocol was approved by the local ethics committees.

*"Definite" PBC*: Three diagnostic criteria were required for definite PBC: (1) a positive IF-AMA test result (≥1 in 80 on Hep2 cells and/or rat tissue slides); (2) pathological serum liver function test(s) [(alkaline phosphatase (ALP) and/or gamma glutamyl transferase (GGT)]; (3) a diagnostic or compatible liver histology (Long *et al.*, 1977).

*"Probable" PBC*: PBC was defined as probable if two of the previous criteria were fulfilled.

*"PBC-AIH overlap" syndrome*: An "overlap" syndrome of PBC and AIH was defined by the simultaneously association of PBC and AIH. The first three criteria for PBC were as above described and the fourth criterion was a serum immunoglobulin M (IgM) level at least 1.5 times above the upper limit of normal value. Criteria for AIH were: (1) serum alanine (ALT) or aspartate (AST) aminotransferase levels at least three times the upper limit of normal; (2) serum immunoglobulin G (IgG) levels at least two times the upper limit of normal values; (3) positive test result(s) for smooth muscle antibodies (SMA) (IF ≥1:80) or anti-nuclear antibodies (ANA) (IF ≥1:80); (4) liver histology showing interface hepatitis. Definite diagnosis of each condition required the presence of at least three of the four criteria.

In each patient, absence of biliary obstruction was assessed by ultrasound. None of the patients had a history of excessive alcohol consumption (>30 g/d), there was no evidence of exposure to hepato- or cholangiotoxic drugs. Serological tests for hepatitis B virus infection were negative. Two patients were found to have antibodies against the hepatitis C virus (anti-HCV) and active viral replication. One of them developed cholestatic liver disease after receiving interferon alfa2a and met all criteria necessary for "definite" PBC. The other had laboratory evidence of cholestasis and histology consistent with PBC.

### Indirect Immunofluorescence Analysis

Sera were diluted 1:40, 1:80 and 1:160 in phosphate buffered saline without magnesium and calcium (PBS). Frozen cryosections of 4 μm-tissue from rat liver, kidney and stomach and HEp2 cell monolayers on glass slides (Labor Diagnostika, Heiden, Germany) were incubated with diluted sera for 30 min at room temperature. Following two washing steps with PBS, the slides were incubated in a humidified, dark chamber for 30 min with fluorescein isothiocyanate-conjugated affinity purified goat anti-human polyclonal (IgG, IgM, IgA) antiserum (Dianova, Hamburg, Germany). Following two washing steps with PBS, tissue slices were analyzed using an Olympus IMT 2 immunofluorescence microscope (Tokyo, Japan). Sera were considered AMA positive with a titer higher that 40.

### Western Blot Analysis

All sera were analyzed by (Western) immunoblotting (WB). Mitochondrial fractions of rat liver cell homogenate were obtained by differential centrifugation. The mitochondrial fraction was subjected to electrophoresis on a sodium-dodecyl sulfate-polyacrylamide gradient (5–20%) gel-electrophoresis (SDS-PAGE) and subsequently transferred to nitrocellulose filter (Trans-Blot, Bio-Rad Labs, Richmond, CA). The blot was stored at $-20°$ C. The strips were rehydrated in PBS and blocked for 20 min with PBS containing 2% (v/w) milk powder. Serum samples were diluted 1:100 in PBS. After removal of the blocking buffer, membrane strips were incubated in the diluted serum for 2 h at room temperature. During the incubation step all specifically bound antibody was detected by alkaline phosphatase-conjugated affinity purified goat anti-human IgA + IgG + IgM (H + L) antibody (Jackson ImmunoResearch Labs, West Grove, PA). After three washings with Tris buffered saline including Tween 20 (0.1%) (TBST) to remove excess enzyme label, specifically bound antibody was detected by the use of a chromogenic reaction with 5′bromo-4′chloro-3′indolyl-phosphate-toluidine and 4′nitro-tetrazolium-chloride Blue (Sigma GmbH, Deisenhofen, Germany) for 15 min.

### Competitive ELISA

A competitive ELISA was used to detect autoantibodies to BCKADC-E2. Briefly, the wells of a 96-round-bottom-well polystyrane plate (Dynatech, Germany) were coated overnight at 4°C with 50 μl of purified IgG BCKADC-E2 antibodies (20 μg protein/ml in PBS) obtained from sera monospecific to anti-BCKADC-E2 as described previously (Long *et al.*, 1977). After removal of the excess autoantibody and washing with PBS with 0.1% Tween 20 (PBS-Tween 20), the free binding sites on the plates were blocked by addition of 1% bovine serum albumin (BSA) in PBS (BSA-PBS) and incubated for 24 h. After washing the wells with PBS-Tween 20, 50 μl of differentially centrifuges mitochondrial protein from rat liver cell homogenate (100 μg/ml in PBS) was added and incubated for 1 h at room temperature on a microplate rotating platform. Plates were washed twice with PBS-Tween 20 before 50 μl of 1:10 diluted serum samples in PBS with 10 mM EDTA were added and incubated for 1 h at room temperature on a rotating platform. In each assay, a strong positive and several negative sera as controls were included and serum samples were measured in duplicate. After washing three times with PBS-Tween 20, 50 μl of the biotin-labeled BCKADC-E2 antibodies (diluted to 1:100 in BSA-PBS) were added to each well. Incubation for 1 h on a microplate rotating platform was followed by three washing steps with PBS-Tween 20. Afterwards, 50 μl of avidin-horseradish peroxidase conjugate (diluted to 1:1500 in BSA-PBS) (Dako, Danemark) were added and incubated for 1 h. After washing the plates, 50 μl of substrate solution (2,2′ azinobis-3-ethylbenzthiazolin sulfonic acid and sodium perborate in citrate buffer) were added. The plates were incubated at room temperature until a dark-color reaction developed in control sera without anti-BCKADC-E2 antibodies. The reaction was stopped by adding 100 μl 1 M citric acid. The optical density was read at 405 nm. Results were expressed as percentage of inhibition of biotinylated reference antibodies to BCKADC-E2. Samples with values higher than the mean value of healthy controls plus 3SD were considered positive. The cut-off for positivity was 20% inhibition of binding.

### Complementary DNA Clones

The complete cDNA of dihydrolipoamide acyltransferase (the E2 subunit) of the human BCKADC-E2 (Gene-Bank/EMBL Data Bank; accession number: J04723) (pkkhE2.gck, the mature protein) was obtained from D.T. Chuang, Texas University, TX 18. The pkkhE2.gck plasmid vector was cut with restriction enzymes *Nco* I and *Eco* RI, and the insert was ligated into the *Nco* I (5′-end) and *Eco* RI (3′-end) restriction sites of pCITE-4a(+) translation enhancer plasmid vector (Novagen, Madison, WI). This construct was designated $pBCKADC_{mat}$ and transformed into competent *Escherichia coli* DH5α cells (Gibco BRL, Eggenstein, Germany). Recombinant clones encoding amino acid residues 1–63 ($pBCKDAC_{Xba\ I}$), 1–76 ($pBCKADC_{Acc\ I}$), 1–144 ($pBCKADC_{Eco\ RV}$) and 1–302 ($pBCKADC_{Bgl\ II}$) were generated by digesting the $pBCKADC_{mat}$ vector with *Xba* I and *Spe* I, *Acc* I, *Eco* RV and *Pme* I and *Bgl* II, respectively. Further, two subclones encoding amino acid residues 1–97 ($pBCKADC_{BspHI}$) and

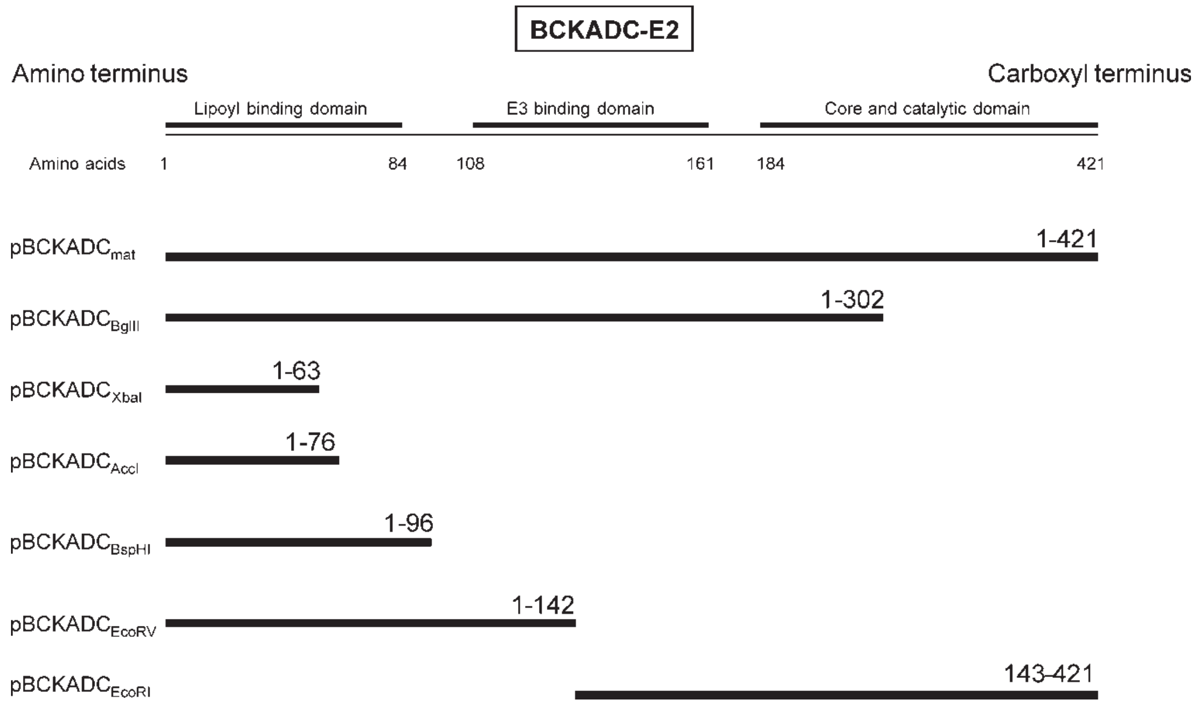

FIGURE 1 Subclones of the complete cDNA encoding the human BCKADC-E2 and its truncated fragments.

143–421 (pBCKADC$_{Eco\ RI}$) were constructed either from pkkhE2.gck vector digested with *Nco* I and *Bsp* HI or from the pBCKADC$_{mat}$ by digesting the clone with *Eco* RV and *Eco* RI. After gel purification, the respective insert was subcloned into pCITE 4a (+) using either *Nco* I or *Eco* RV and *Eco* RI (Fig. 1). The identity of the plasmids was controlled by sequencing using the dideoxy sequencing method according to the manufacturer's specifications (Sanger *et al.*, 1990).

### *In Vitro* Transcription/*In Vitro* Translation of the $^{35}$S-labeled BCKADC-E2 Products

*In vitro* transcription and *in vitro* translation of 2 μg of pBCKADC$_{mat}$ and its subclones were performed by the TNT T7 Quick Coupled Transcription/Translation System from rabbit reticulocyte lysate (Promega, Madison, WI) in a volume of 50 μl at 30°C for 90 min in the presence of 2 mCi/μl of ($^{35}$S)-methionine (Amersham, UK). The amount of ($^{35}$S)-labeled BCKADC-E2 and its truncated fragments was quantified after SDS-PAGE using a Fuji Bas 1000 bioimaging analyzer system (Raytest, Straubenhardt, Germany).

### Radioimmunoprecipitation Assay

For quantitative antibody assay, 50,000 cpm of the pooled ($^{35}$S)-methionine-labeled protein was incubated over night at 4°C with 1 μl of serum tested in a total volume of 50 μl WP0 buffer (20 mM Tris–HCl (pH 7.4), 150 mM NaCl, 0.5% Tween 20) on a rotating platform. Antibody-bound labeled protein was isolated with 1 mg protein A (Affi-Prep A, Bio-Rad Lab, Hercules, CA) (Hjelm *et al.*, 1975). After incubation for 30 min at 4°C a total of 75 μl immunoprecipitation mixture was washed two times with 750 μl of WP1 buffer (20 mM Tris–HCl (pH 7.4), 150 mM NaCl, 0.5% Tween 20, 0.1% BSA). The last wash step was performed with WP2 buffer (50 mM Tris–HCl (pH 6.8), 0.1% Tween 20). Immune complexes captured by protein A were boiled for 5 min in SDS-sample buffer. Undissolved components were removed by centrifugation. Ten microliters from supernatant was diluted in 1 ml scintillation fluid (Optiphase Supermix, Wallac, Turku, Finland), and the amount of radiolabeled protein was determined with a 1450 Micro Beta Tailux Counter (Wallac, Turku, Finland). Twenty sera recognizing both N- and C-terminal sequences of the enzyme were titered to the end dilution. Each serum was assayed in duplicate in separate experiments, and the binding of different BCKADC-E2 protein fragments was reported as the mean of two assays. A mean interassay difference was between 13% and 19%.

### RESULTS

### Patients and Sera

Of the 96 IF-AMA-positive sera evaluated by WB and ELISA, 15 reacted only with PDC-E2 and 81 recognized BCKADC-E2 which were used to map autoepitopes to human BCKADC-E2. Of those 81 sera, 26 were immunoreactive only with BCKADC-E2 and 55 sera had autoantibodies to both BCKADC-E2 and PDC-E2. Of the 81 patients, 68 met diagnostic criteria sufficient

TABLE I Immunoreactivity of sera against BCKADC-E2, N- and C-terminal sequences of the mature protein

| Patients | | Mature BCKADC-E2 | N-terminal (1–144 aa) | C-terminal (143–421 aa) |
|---|---|---|---|---|
| Controls | (*n*=94) | | | |
| Blood donors | (*n*=30) | 0 | 0 | 0 |
| AIH | (*n*=10) | 2* | 0 | 0 |
| PSC | (*n*=14) | 0 | 0 | 0 |
| Rheumatoid arthritis | (*n*=40) | 0 | 0 | 0 |
| Anti-PDC-E2+ PBC Anti-BCKADC-E2+ | (*n*=15) | 0 | 0 | 0 |
| Definite PBC | (*n*=66) | 66 | 56 | 44 |
| Without OLT | (*n*=54) | 54 | 45 | 39 |
| Post-OLT | (*n*=12) | 12 | 11 | 5 |
| Probable PBC | (*n*=2) | 2 | 2 | 2 |
| “PBC-AIH overlap” syndrome | (*n*=7) | 7 | 6 | 1 |
| Undetermined case | (*n*=5) | 5 | 0 | 0 |

*Abbreviations*: BCKADC-E2, branched-chain acyltransferase; AIH, autoimmune hepatitis; PSC, primary sclerosing cholangitis; PDC-E2, dihydrolipoamide acetyltransferase; OLT, orthotopic liver transplantation; PBC, primary biliary cirrhosis; *, borderline results.

for a “definite” ($n = 66$) or a “probable” ($n = 2$) diagnosis of PBC. Twelve PBC sera were obtained from patients that had undergone orthotopic liver transplantation (OLT). The median post-OLT time was 5.2 years (range 1–12 years). Two patients developed the defined criteria of PBC after a 6-month course of interferon alfa2a therapy. The IF analysis of PBC sera revealed 32/68 (47%) ANA positive cases. Patterns evaluated on HEp2 cells included multiple nuclear dots ($n = 13$), speckled ($n = 10$), centromere ($n = 5$), rim-like ($n = 3$) and nucleolar ($n = 1$) features. Antibodies against smooth-muscle cells (SMA) were detected in three sera. Seven of 81 patients met diagnostic criteria of an “PBC-AIH overlap” syndrome. ANA positivity was observed in six cases and one serum had SMA reactivity. Of the 81 evaluated cases five did not have enough available data to make a definite diagnosis (Table I).

### Sensitivity and Specificity of the Anti-BCKADC-E2 Radioligand Assay

Our radioimmunoassay was established and found capable of measuring autoantibodies reactive with the BCKADC-E2 target autoantigen. The RLA is so far the first assay utilizing human recombinant ($^{35}$S)-labeled BCKADC-E2 protein which was produced by *in vitro* transcription/*in vitro* translation. Eighty of 81 sera positive for BCKADC-E2 by WB and ELISA were reactive with the human BCKADC-E2. Figure 2 shows that the identified positive sera were above the cut-off value of 612 cpm (the median value of 30 healthy control samples plus 3SD). The mean value of the BCKADC-E2 positive sera was 8176 cpm (range 644–34,580 cpm). Of the IF-AMA- and BCKADC-E2-positive sera only one gave a weak reactivity with the mature protein (value 644 cpm). There was no difference among sera recognizing only BCKADC-E2 and those with reactivity toward both PDC-E2 and BCKADC-E2.

None of sera from patients with PSC or rheumatoid arthritis reacted with the human BCKADC-E2. Of the AIH sera two showed a slightly elevated immunoreactivity above the cut-off level (666 and 626 cpm, respectively). Both of them were IF-AMA-negative and there was no evidence of bile duct involvement. No serum recognizing only PDC-E2 was immunoreactive with BCKADC-E2 indicating the lack of cross-reactivity between

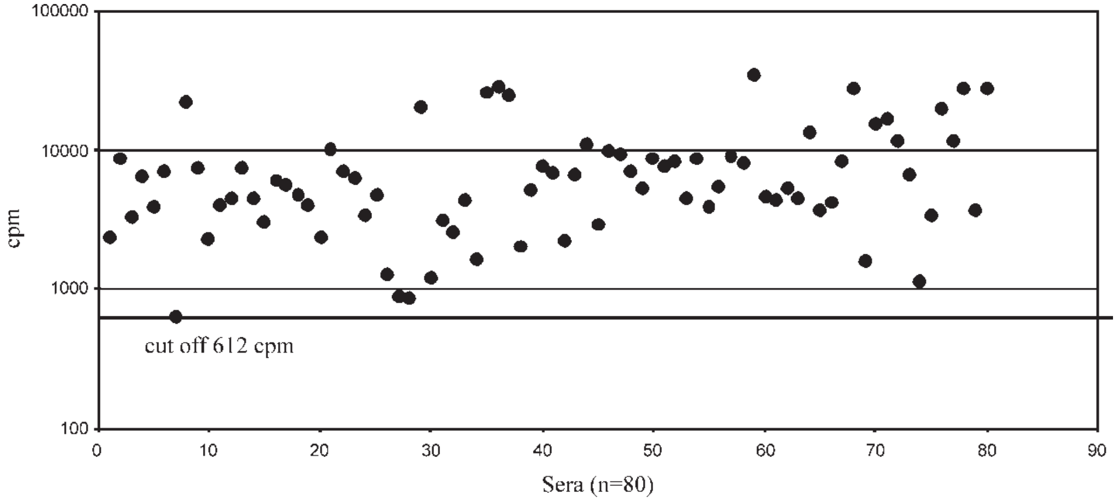

FIGURE 2 Reactivity of BCKADC-E2 positive sera with the human BCKADC-E2 by radioimmunoprecipitation assay.

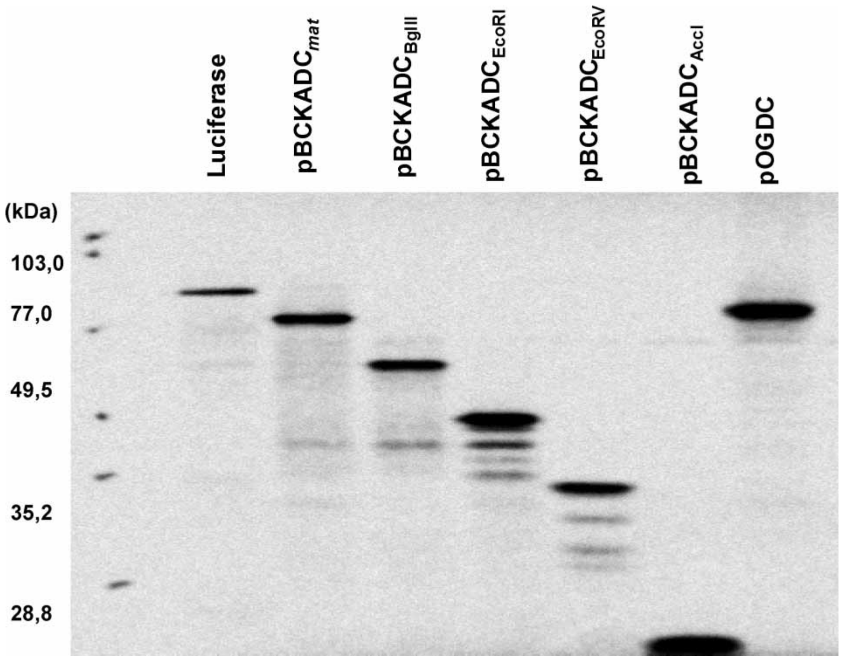

FIGURE 3 $^{35}$S-labeled *in vitro* translation products of human BCKADC-E2 cDNA clones expressed in rabbit reticulocyte lysate and evaluated in 12% SDS-PAGE and by bioimaging analyzer.

autoantibodies specific either to BCKADC-E2 or to PDC-E2 as immune target. Based on our analysis the sensitivity and specificity of this assay was determined to be 99 and 98%, respectively.

### Epitope Mapping Experiments

All cDNA sequences subcloned into pCITE expression vector encoding fragments of the human BCKADC-E2 were expressed in an *in vitro* translation/*in vitro* transcription system and visualized by 12% SDS-PAGE and phosphoimaging analyzer (Fig. 3). The characteristics of expressed protein products and results of epitope mapping experiments are shown in Table II. Thirty two to 34 amino acid residues encoded by pCITE vector sequence were added to the cDNA encoded sequences in the vector. The discrepancy between the molecular mass calculated from the deduced primary sequence of BCKADC-E2 protein products and that determined by SDS-PAGE is the result of retarded migration of the lipoyl-bearing domain by SDS-PAGE (Bleile *et al.*, 1981; Guest *et al.*, 1985).

The smallest fragment of BCKADC-E2 which was detected by 63% of the evaluated sera required amino acid residues 1–76. The vast majority of sera displayed immunoreactivity toward two clones encoding either residues 1–97 (pBCKADC$_{Bsp\ \mathrm{HI}}$) and residues 1–302 (pBCKADC$_{Bgl\ \mathrm{II}}$) (Table II). Nine of 81 sera recognized only the mature protein. Of particular interest was that 47 of 80 sera displayed strong reactivity toward the C-terminal part of BCKADC-E2 (residues 143–421) encoded by pBCKADC$_{Eco\ \mathrm{RI}}$. Figure 4 shows that about two thirds of PBC sera recognized C-terminal sequences of the immune target, and some sera showed considerable reactivity with the C-terminal domain. Three sera exclusively recognized the C-terminus of BCKADC-E2 and displayed no immunoreactivity toward proteins encompassing lipoyl binding domain. N-terminal sequences encoded by plasmid pBCKADC$_{Eco\ \mathrm{RV}}$ were recognized by 85% of the sera. PBC sera yielded reactivity with N-terminal sequences in 85% and with C-terminal sequences in 68%. Eleven of 12 sera from post-OLT PBC patients were reactive with the lipoyl binding domain. However, the C-terminus of BCKADC-E2 was

TABLE II Characteristics of recombinant proteins encoded by BCKADC-E2 cDNA clones and their reactivity with sera ($n = 80$) recognizing the branched-chain acyltransferase

| Clone | Amino acid residues | Number of $^{35}$S-methionine | Cut-off level (cpm) | Reactive sera (%) |
|---|---|---|---|---|
| pBCKADC$_{\mathrm{mat}}$ | (32) + 1–421 | 15 (3* + 12) | 612 | 100 |
| pBCKADC$_{\mathrm{XbaI}}$ | (32) + 1–67 | 4 (3* + 1) | 285 | 0 |
| pBCKADC$_{\mathrm{AccI}}$ | (32) + 1–76 | 4 (3* + 1) | 479 | 63 |
| pBCKADC$_{\mathrm{BspHI}}$ | (32) + 1–97 | 4 (3* + 1) | 279 | 85 |
| pBCKADC$_{\mathrm{EcoRV}}$ | (32) + 1–144 | 5 (3* + 2) | 294 | 80 |
| pBCKADC$_{\mathrm{BglII}}$ | (32) + 1–302 | 11 (3* + 8) | 465 | 85 |
| pBCKADC$_{\mathrm{EcoRI}}$ | (34) + 143–421 | 12 (4* + 10) | 613 | 59 |

*Methionine residues encoded by pCITE plasmid vector.

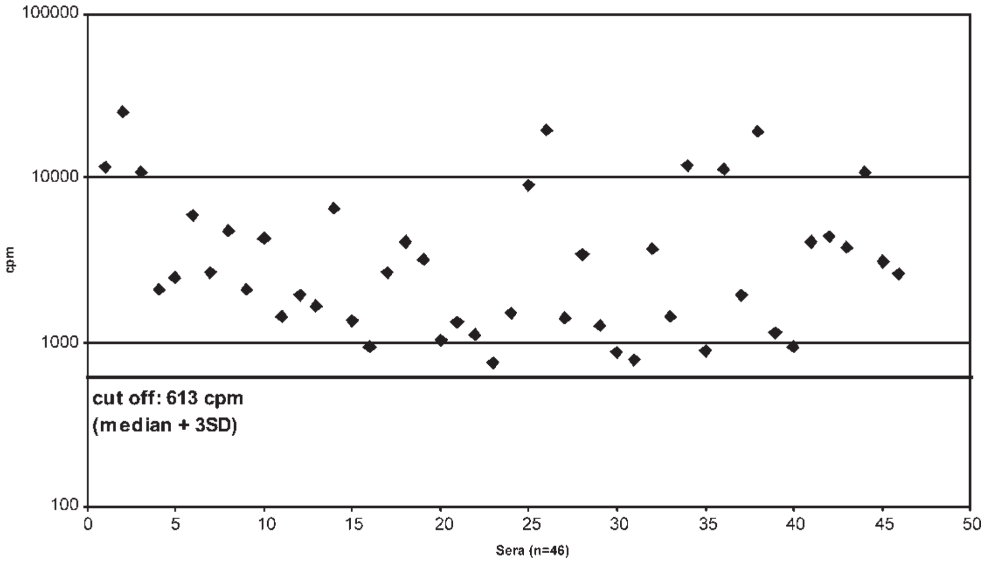

FIGURE 4 Immunoreactivity of BCKADC-E2 positive sera against C-terminal sequences of the human branched chain acyltransferase.

recognized only by 5/12 sera from patients who had OLT. Of "PBC-AIH overlap" sera ($n = 7$) only one recognized C-terminal sequences on the human BCKADC-E2. There was no difference between sera obtained from Hungarian or German patients (Table I). Neither the clinical course nor biochemical and histological data indicated any significant differences between PBC patients whose sera recognized C-terminal sequences of BCKADC-E2 and those who did not. In our RLA, Protein A was used to capture immunocomplexes consisting of AMA and radioactive-labeled proteins. Human IgG1, 2 and 4 bind strongly to Protein A, while IgG3 and IgM isotypes do not bind to it (Guest *et al.*, 1985). Ninety two percent of sera tested were immunoreactive with the human, recombinant unlipoylated BCKADC-E2. These data indicate no notable restriction to the IgG3 and IgM subclasses as was reported on AMA specific to PDC-E2 (Surh *et al.*, 1988). Furthermore, lipoylation of the immune target is not essential for recognition by AMA specific to BCKADC-E2 as reported in connection with PDC-E2 (Fussey *et al.*, 1989).

### Titer of Autoantibodies Recognizing N- and C-terminal Sequences of BCKADC-E2

Immunoprecipitation of recombinant BCKADC-E2 protein products (mature BCKADC-E2, N-terminal domain of 1–144 aa and C-terminal sequences of 143–421 aa) was performed with various serum dilutions. Twenty PBC sera recognizing both N- and C-terminal parts of the protein were used for further studies. The baseline dilution was 1:50. While at a dilution of 1:500 17 sera reacted with the studied protein products, in a titer of 5000, 60% (12/20) of the tested sera recognized both C- and N-terminal sequences. At a dilution of 1:50,000 two sera showed a weak reactivity with the C-terminal part of BCKADC-E2, and no serum recognized N-terminal sequences.

### DISCUSSION

Antibodies in response to an antigenic stimulation are polyclonal and they recognize multiple epitopes on immune targets (Benjamin *et al.*, 1984; Laver *et al.*, 1990). Based on molecular modeling studies of antibodies reacting with (auto)antigens at least 90% of B cell (auto)epitopes are believed to be conformational rather than sequential and linear (Blundell *et al.*, 1987). Evidence obtained by epitope mapping of a number of autoantigens suggests that the antibody response in autoimmune diseases is comparable to that occurring under conditions of infection. In contrast to T cell autoepitopes which are represented as short linear peptides, immunodominant epitopes for B cells are more complex and frequently involve functionally essential sites of the target molecules (Whittingham and McNeilage, 1988; Galperin and Gershwin, 1996). AMA occurring in PBC sera share several similarities with those that are present in other autoimmune diseases such as in systemic lupus erythematodes or in scleroderma. However, in contrast to autoantibodies in systemic lupus erythematodes or Sjögren's syndrome which recognize multiple epitopes on their immune target, it is an unusual feature of PBC-specific AMA that only one major conformational epitope has been reported on all target mitochondrial enzymes which encompasses the lipoyl binding domain (Tan, 1989).

The usual method for mapping B cell autoepitopes to immune targets is by WB which preferentially detects linear epitopes resulting from linear sequences exposed by the unfolding of the protein. However, the WB tends to destroy many antibody binding sites created by a three-dimensional folding of the protein. Therefore, important conformational epitopes may be missed when this method is exclusively employed for epitope mapping analyses. It is therefore possible that components recognized by WB may be pseudoepitopes arising from linear sequences exposed by the unfolding of the protein during denaturation (Rowley *et al.*, 1991). Many of the data on autoepitopes recognized by AMA in PBC were obtained from studies in which WB was employed for epitope mapping.

Evidence is increasing that the radioimmunoprecipitation assay using unmodified recombinant proteins allows for the detection of high-affinity autoantibodies directed against both linear and conformational sequences of autoantigens in addition of providing a highly sensitive method for autoantibody quantification. An example are the conformational epitopes located on the proliferating cell nuclear antigen (PCNA) which were not detectable by WB, but by immunoprecipitation assay with a full-length construct of the antigen (Huff *et al.*, 1990). However, a potential limitation of the assay, is the potential failure to detect autoantibodies directed against post-translationally modified epitopes (e.g. lipoylation, acetylation).

BCKADC-E2 has been implicated as an immune target in two diseases: idiopathic dilatativ cardiomyopathy (Ansari *et al.*, 1994) and PBC (Fregeau *et al.*, 1989; James *et al.*, 1989; Surh *et al.*, 1989; Leung *et al.*, 1995). By using a full-length cDNA clone for BCKADC-E2 and truncated mutants, the immunodominant epitope recognized by sera from patients with idiopathic dilatative cardiomyopathy was localized to amino acid residues 116–134 corresponding to the E3-binding domain (Fig. 1), and these sera inhibited the enzyme activity (Ansari *et al.*, 1994). In contrast to this finding, the epitope in PBC appears to be dependent on the three dimensional protein structure and to include the lipoic acid binding region (residues 1–115). Antibody binding to the lipoyl-binding region is not dependent on the presence of lipoic acid (Leung *et al.*, 1995).

Of particular interest was that only the full-length clone (amino acid residue 1–421) was sufficient to remove all detectable BCKADC-E2 reactivity. The authors suggested that the inner E2 core and the E3 binding domain by themselves did not have any antibody binding (Leung *et al.*, 1995).

Our results indicate a novel, highly sensitive and specific RLA using ($^{35}$S)-labeled human branched-chain acyltransferase to detect autoantibodies specific to BCKADC-E2. Of the sera positive for anti-BCKADC-E2 by WB and ELISA, 99% recognized the human mature BCKADC-E2 protein by RLA. This assay provides a sensitive and specific tool for the quantification of the autoantibody response toward BCKADC-E2 in autoimmune liver diseases. Our data confirmed the findings reported by Leung *et al.* (1995) that an immunodominant epitope resided on the N-terminal part of BCKADC-E2, and antibody binding did not require the presence of lipoic acid. The lack of immunoreactivity of sera against the N-terminal three-fourth of lipoyl binding domain (residues 1–63) showed that N-terminal epitope was conformational and additional sequences of the first hinge region were also required.

In addition to the previous finding this study showed a novel, major B cell autoepitope residing on the C-terminal domain of BCKADC-E2 which was recognized by approximately 70% of sera from patients with PBC. Reactivity directed against C-terminal sequences was independent from reactivity measured in the same sera againts lipoyl binding domain. Some sera contained AMA to C-terminal sequences of BCKADC-E2 in higher titer than those recognizing lipoyl binding domain. Of particular interest was that sera derived from patients presenting with an ”overlap” syndrome of PBC and AIH recognized the mature BCKADC-E2 and N-terminal epitope, but not the C-terminal sequences of the autoantigen. A degree of heterogeneity of the immune response to BCKADC-E2 in PBC is illustrated by our findings that 11% of sera exclusively recognized the mature protein, and some sera were reactive only with C-terminal sequences.

The binding property of Protein A permits the formation of tertiary complexes consisting of protein A, antibody and antigen (Goding, 1978). In our reported RLA using Protein A, 99% of sera recognized the human, unlipoylated enzyme indicating that BCKADC-E2-specific AMA response is not restricted to IgM and IgG3.

In conclusion, we established an immunoprecipitation-based detection assay using recombinant human BCKADC-E2 protein. We identified a novel B cell autoepitope on C-terminal part of the human branched-chain acyltransferase. N- and C-terminal sequences of BCKADC-E2 were recognized at comparable frequences (85 and 68%, respectively) by PBC sera. Our data indicate that a distinct subset of AMA recognize sequence(s) on BCKADC-E2 which are independent of the lipoyl binding domain. Because sera from patients presenting features of two autoimmune liver diseases, notably PBC and AIH, did not recognize C-terminal sequences, the lack of immunoreactivity to the C-terminal domain may have diagnostic importance in distinguishing PBC patients from those with “overlap” syndrome. Further studies are required to obtain more relevant data to confirm the clinical significance of this new finding.

***Acknowledgements***

A. Csepregi was supported by a research grant from the European Association for the Study of the Liver. This work was further supported by the Deutsche Forschungsgemeinschaft SFB 244-C11 and by the Hungarian Scientific Research Fund No F026455. PD Dr Strassburg is a Heisenberg scholar of the Deutsche Forschungsgemeinschaft.

### *References*

Ansari, A.A., Neckelmann, N., Villinger, F., *et al.* (1994) "Epitope mapping of the branched chain α-keto acid dehydrogenase dihydrolipoyl transacylase (BCKD-E2) protein that reacts with sera from patients with idiopathic dilated cardiomyopathy", *J. Immunol.* **153**, 4754–4765.

Arnett, F.C., Edworthy, S.M., Bloch, D.A., *et al.* (1988) "The American Rheumatism Association 1987 revised criteria for the classification of rheumatoid arthritis", *Arthritis Rheum.* **31**, 315–324.

Baum, H. (1994) In: Lee, C.P., ed, Molecular Aspects of Mitochondrial Pathology Current Topics in Bioenergetics, Vol. **17**, pp 127–171.

Benjamin, D.C., Berzofsky, J.A., East, I.J., *et al.* (1984) "The antigenic structure of proteins: a reappraisal", *Annu. Rev. Immunol.* **2**, 67–101.

Berg, P. and Klein, R. (1995) "Mitochondrial antigen/antibody systems in primary biliary cirrhosis: revisited", *Liver* **15**, 281–292.

Bleile, D.M., Hackert, M.L., Pettit, F.H. and Reed, L.J. (1981) "Subunit structure of dihydrolipoyl transacetylase component of pyruvate dehydrogenase complex from bovine heart", *J. Biol. Chem.* **256**, 514–519.

Blundell, T.L., Sibanda, B.L., Sternberg, M.J. and Thornton, J.M. (1987) "Knowledge-based prediction of protein structures and the design of novel molecules", *Nature* **326**, 347–352.

Burroughs, A.K., Butler, P., Sternberg, M.J.E. and Baum, H. (1992) "Molecular mimicry in liver disease", *Nature* **358**, 377–378.

Coppel, R.L., McNeilage, L.J., Surh, C.D., *et al.* (1988) "Primary structure of the human M2 mitochondrial autoantigen of primary biliary cirrhosis: dihydrolipoamide acetyltransferase", *Proc. Natl Acad. Sci. USA* **85**, 7317–7321.

Fregeau, D.R., Davis, P.A., Danner, D.J., *et al.* (1989) "Anti-mitochondrial antibodies of primary biliary cirrhosis recognize dihydrolipoamide acyltransferase and inhibit enzyme function of the branched chain α-keto acid dehydrogenase complex", *J. Immunol.* **142**, 3815–3820.

Fussey, S.P.M., Bassendine, M.F., James, O.F.W. and Yeaman, S.J. (1989) "Characterisation of the reactivity of autoantibodies in primary biliary cirrhosis", *FEBS Lett.* **246**, 49–53.

Galperin, C. and Gershwin, M.E. (1996) "Immunopathology of primary biliary cirrhosis", *Bailliere's Clin. Gastroenterol.* **10**, 461–481.

Gershwin, M.E., Mackay, I.R., Sturgess, A. and Coppel, R.L. (1987) "Identification and specificity of a cDNA encoding the 70 kD mitochondrial antigen recognized in primary biliary cirrhosis", *J. Immunol.* **138**, 3525–3531.

Goding, J.W. (1978) "Use of staphylococcal protein A as an immunological reagent", *J. Immunol. Methods* **20**, 241–253.

Griffin, T.A., Wynn, R.M. and Chuang, D.T. (1990) "Expression and assembly of mature apotransacylase (E2b) of bovine branched chain α-keto acid dehydrogenase complex in *Escherichia coli*", *J. Biol. Chem.* **265**, 12104–12110.

Guest, J.R., Lewis, H.M., Graham, L.D., *et al.* (1985) "Genetic reconstruction and functional analysis of the repeating lipoyl domains in the pyruvate dehydrogenase multienzyme complex of *Escherichia coli*", *J. Mol. Biol.* **185**, 743–754.

Hjelm, H. (1975) "Isolation of IgG3 from normal sera and from a patients with multiple myeloma by using protein A-sepharose 4B", *Scand. J. Immunol.* **4**, 633–640.

Huff, J.P., Roos, G., Peebles, C.L., *et al.* (1990) "Insights into native epitopes of proliferating cell nuclear antigen using recombinant DNA protein products", *J. Exp. Med.* **172**, 419–429.

Iwayama, T., Leung, P.S.C., Rowley, M., *et al.* (1992) "Comparative immunoreactive profiles of Japanese and American patients with primary biliary cirrhosis against mitochondrial autoantigens", *Int. Arch. Allergy Immunol.* **99**, 28–33.

James, O.F.W., Yeaman, S.J. and Bassendine, M.F. (1989) "Molecular aspects of the M2 autoantigens in primary biliary cirrhosis: what a difference a year makes", *Hepatology* **10**, 247–251.

Johnson, P.J. and McFarlane, I.G. (1992) "International Autoimmune Hepatitis Group Meeting Report. Autoimmune hepatitis: criteria for diagnosis and response to therapy", *Hepatology* **18**, 998–1006.

Kaplan, M.M. (1996) "Primary biliary cirrhosis", *N. Engl. J. Med.* **335**, 1570–1580.

Kim, W.R., Lindor, K.D., Locke, III, G.R., *et al.* (2000) "Epidemiology and natural history of primary biliary cirrhosis in a US community", *Gastroenterology* **119**, 1631–1636.

Lau, K.S., Chuang, J.L., Herring, W.J., *et al.* (1992) "The complete cDNA sequence for dihydrolipoyl transacylase (E2) of human branched-chain alpha-keto acid dehydrogenase complex", *Biochim. Biophys. Acta* **1132**, 319–321.

Laver, W.G., Air, G.M., Webster, R.G. and Smith-Gill, S.J. (1990) "Epitopes on protein antigens: misconceptions and realities", *Cell* **61**, 553–556.

Leung, P.S.C., Chuang, D.T., Wynn, R.M., *et al.* (1995) "Autoantibodies to BCOADC-E2 in patients with primary biliary cirrhosis recognize a conformational epitope", *Hepatology* **22**, 505–513.

Long, R.G., Scheuer, P.J. and Sherlock, S. (1977) "Presentation and course of asymptomatic primary biliary cirrhosis", *Gastroenterology* **72**, 1204–1207.

Migliaccio, Ch., Nishio, A., van de Water, J., *et al.* (1998) "Monoclonal antibodies to mitochondrial E2 components define autoepitopes in primary biliary cirrhosis", *J. Immunol.* **161**, 5157–5163.

Rowley, M.J., McNeilage, L.J., Armstrong, J.M. and Mackay, I.R. (1991) "Inhibitory autoantibodies to a conformational epitope of the pyruvate dehydrogenase complex, the major autoantigen in primary biliary cirrhosis", *Clin. Immunol. Immunopathol.* **60**, 356–370.

Sanger, F., Nicklen, S. and Coulson, A.R. (1990) "DNA sequencing with chain terminating inhibitors", *Proc. Natl Acad. Sci. USA* **74**, 5463–5467.

Surh, C.D., Cooper, A.E., Coppel, R.L., *et al.* (1988) "The predominance of IgG3 and IgM isotype antimitochondrial antibodies against recombinant fused mitochondrial polypeptide in patients with primary biliary cirrhosis", *Hepatology* **8**, 290–295.

Surh, C.D., Danner, D.J., Ansari, A.A., *et al.* (1989) "Reactivity of primary biliary cirrhosis sera with a human fetal liver cDNA clone of branched α-keto acid dehydrogenase dihydrolipoamide acyltransferase, the 52 kD mitochondrial autoantigen", *Hepatology* **9**, 63–68.

Surh, C.D., Coppler, R.L. and Gershwin, M.E. (1990) "Structural requirement for autoreactivity on human PDH-E2, the major autoantigen of primary biliary cirrhosis: implication for a conformational autoepitope", *J. Immunol.* **144**, 3367–3374.

Tan, E.M. (1989) "Antinuclear antibodies: diagnostic markers for autoimmune diseases and probes for cell biology", *Adv. Immunol.* **44**, 93–151.

Van de Water, J., Gershwin, M.E., Leung, P., *et al.* (1988) "The autoepitope of the 74 kD mitochondrial autoantigen of primary biliary cirrhosis corresponds to the functional site of dihydrolipoamide acetyltransferase", *J. Exp. Med.* **167**, 1791–1799.

Walker, J.G., Doniach, D., Roitt, I.M. and Sherlock, S. (1965) "Serological tests in diagnosis of primary biliary cirrhosis", *Lancet* **i**, 827–831.

Whittingham, S. and McNeilage, L.J. (1988) "Antinuclear antibodies as molecular and diagnostic probes", *Mol. Cell Probes* **2**, 169–179.

Wiesner, R.H. and LaRusso, N.F. (1980) "Clinicopathologic features of the syndrome of primary sclerosing cholangitis", *Gastroenterology* **79**, 200–206.

